# Genomic deletions in *OPA1 *in Danish patients with autosomal dominant optic atrophy

**DOI:** 10.1186/1471-2350-12-49

**Published:** 2011-04-04

**Authors:** Gitte J Almind, Karen Grønskov, Dan Milea, Michael Larsen, Karen Brøndum-Nielsen, Jakob Ek

**Affiliations:** 1 Center for Applied Human Molecular Genetics, The Kennedy Center, Glostrup, Denmark; 2 Ophthalmology Department, Angers University Hospital, Angers, France; 3 Department of Ophthalmology, Glostrup Hospital, Glostrup, Denmark; 4 Faculty of Health Sciences, University of Copenhagen, Copenhagen, Denmark; 5 Department of Clinical Genetics, Rigshospitalet, Copenhagen, Denmark

## Abstract

**Background:**

Autosomal dominant optic atrophy (ADOA, Kjer disease, MIM #165500) is the most common form of hereditary optic neuropathy. Mutations in *OPA1 *located at chromosome 3q28 are the predominant cause for ADOA explaining between 32 and 89% of cases. Although deletions of *OPA1 *were recently reported in ADOA, the frequency of *OPA1 *genomic rearrangements in Denmark, where ADOA has a high prevalence, is unknown. The aim of the study was to identify copy number variations in *OPA1 *in Danish ADOA patients.

**Methods:**

Forty unrelated ADOA patients, selected from a group of 100 ADOA patients as being negative for *OPA1 *point mutations, were tested for genomic rearrangements in *OPA1 *by multiplex ligation probe amplification (MLPA). When only one probe was abnormal results were confirmed by additional manually added probes. Segregation analysis was performed in families with detected mutations when possible.

**Results:**

Ten families had *OPA1 *deletions, including two with deletions of the entire coding region and eight with intragenic deletions. Segregation analysis was possible in five families, and showed that the deletions segregated with the disease.

**Conclusion:**

Deletions in the *OPA1 *gene were found in 10 patients presenting with phenotypic autosomal dominant optic neuropathy. Genetic testing for deletions in *OPA1 *should be offered for patients with clinically diagnosed ADOA and no *OPA1 *mutations detected by DNA sequencing analysis.

## Background

Autosomal dominant optic atrophy (ADOA) is the most common hereditary optic neuropathy. The phenotype is characterized by bilateral subnormal visual acuity, colour vision defect, a partial or absolute centrocoecal scotoma, optic nerve pallor, and subnormal retinal nerve fiber layer and ganglion cell layer thickness [1,2]. The disease has incomplete penetrance and variable expression, ranging from subclinical visual manifestations to legal blindness [3]. The highly variable phenotype, both within and between pedigrees, suggests that genetic and/or environmental cofactors influence the expression of the disease. Kjer's optic atrophy or optic atrophy 1 (MIM #165500) [4], the ADOA originally described by Kjer, is caused by mutations in *OPA1 *(chromosome 3q28-q29). A specific frameshift mutation in exon 28 is particularly common in Denmark with evidence for a founder effect [5-7]. Other loci for ADOA include *OPA4 *on chromosome 18q12.2-q12.3 [8] and *OPA5 *on chromosome 22q12.1-q13.1 [9]. Dominant mutations in *OPA3 *have been recently reported in ADOA associated with cataract [10]. The gene most commonly involved in ADOA is *OPA1 *[11,12] in which 205 unique pathogenic mutations have been identified http://lbbma.univ-angers.fr/eOPA1[13]. Many mutations have only been found in a single family. The prevalence of *OPA1 *mutations in ADOA patients ranges from 32 to 89%, suggesting the existence of other causative genes or alternative types of genetic defects, including genomic rearrangements [14-18].

Genomic deletions or duplications have been found to account for various genetic disorders [19-21]. Marchbank et al. [22] were first to identify complete deletion of *OPA1 *as a cause of ADOA. Recently, Fuhrmann et al. have shown that genomic aberrations may explain up to 12.9% of cases of Kjer-type ADOA [23].

Because a large fraction of our ADOA cases lacked a molecular diagnosis, in spite of having typical family histories in most cases, we initiated a study of copy number variation and other genomic rearrangements at the *OPA1 *locus to supplement the results of direct sequencing. We investigated 40 index patients diagnosed on clinical grounds with ADOA who had previously been found negative for mutations in *OPA1 *by DNA sequence analysis.

## Methods

### Patients and control subjects

One hundred unrelated index patients, of Danish origin, with clinically diagnosed ADOA were retrieved from the Danish ophthalmogenetics register and DNA repository of the National Eye Clinic at the Kennedy Center. The study included only cases from families with at least two affected members and an autosomal dominant pattern of inheritance. The diagnosis was based on routine clinical procedures, the standard being refraction and determination of best corrected visual acuity, color vision testing, visual evoked potential recording, fundus photography, Goldmann manual kinetic perimetry, and slit-lamp biomicroscopy of the anterior segment, vitreous, and posterior pole. No other inflammatory, ischemic, toxic causes of optic neuropathies were detected. Genomic DNA was obtained from the index patients and first degree relatives when available. Genomic DNA was extracted from leucocytes using Chemagic Magnetic Separation Module I (Chemagen, Baesweiler, Germany). The patients were screened for mutations in all coding regions and exon-intron boundaries by direct sequencing using BigDye chemistry and analyzed using an ABI 3130 instrument (Applied Biosystems, Foster City, CA, USA) (unpublished data). Of the 100 index patients, 40 were not harboring an identifiable *OPA1 *mutation. DNA from these 40 index patients was then analyzed for genomic rearrangements in *OPA1 *using multiplex ligation probe amplification (MLPA). The study was performed in accordance to the Helsinki declaration and was approved by the local ethics committee. Patients and healthy relatives had given their written informed consent.

### MLPA Analysis

MLPA analysis was performed using a commercial kit (P229-B1, MRC-Holland, Amsterdam, The Netherlands) following the manufacturer's instructions. The MLPA KIT P229-B1 contains probes for 30 of 31 exons in *OPA1*. Additional MLPA probes were designed in-house to amplify regions narrowing down the identified deletions and to confirm initial findings. For the reactions we used 150 ng of patient DNA. The reactions were separated and visualized on an ABI 3130 Genetic Analyzer and further analyzed using GeneMarker (SoftGenetics, State College, PA, USA). Patients with all *OPA1 *exons deleted were further analyzed using both the P264 MLPA kit from MRC-Holland containing additional probes in the 3q29 telomere region (including OPA1, GP5, LSG1, CENTB2, TNK2, UBXD7, PAK2, MFI2, DLG1, BDH, KIAA0226, LMLN) and using manually designed probes with the P200 kit from MRC-Holland (probe sequences are available upon request). The results were considered significant when the peak height ratio of the normalized sample compared to the normalized average of controls was above 1.3 or below 0.65.

## Results

**Figure 1:**
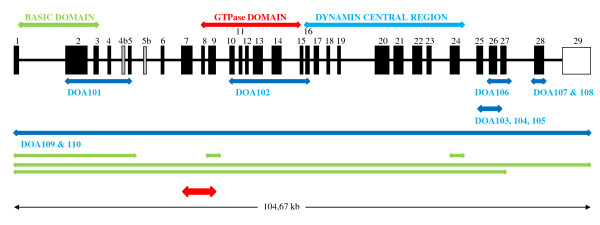
**Deletions and duplications identified in the *OPA1 *gene**. Black boxes illustrate exons and light grey boxes illustrate alternative spliced exons. Arrows above the gene show the functional domains. Arrows below the gene show localization of deletions identified in the present study (blue arrows) and deletions (green arrows) and duplication (red arrow) identified by Fuhrmann et al. (2009) [23].

**Figure 2:**
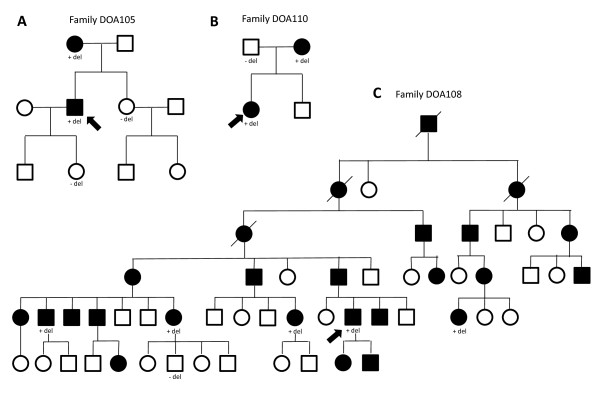
**Pedigrees of three ADOA families A. Family DOA105, B. Family DOA110 C. Family DOA108**. Index patients are indicated with an arrow. Filled symbols are individuals affected with ADOA, open symbols are either unaffected or not known. Individuals investigated by deletion analysis are shown with a "+del" (deletion present) or "-del" (no deletion) below symbol.

We identified 10 index patients with deletions in *OPA1 *(Figure 1 and Additional file 1), all patients being heterozygous. Clinical data are presented in table 1. No rearrangements other than deletions were found. Complete deletion of all 31 *OPA1 *exons was identified in the index patients from families DOA109 and DOA110. Further investigation with MLPA kit P264 showed that the *GP5 *gene, which is located telomeric to *OPA1*, was intact in both cases. Segregation analysis of family DOA110 showed that the deletion segregated with the disease (Figure 2B). Eight intragenic deletions spread across the gene were found in *OPA1*. The most N-terminal deletion was found in index patient from family DOA101 where exons 2-5 were deleted. In the index patient from family DOA102 we found a deletion of exon 10-16. In three independent families (DOA103, DOA104 and DOA105) we identified a deletion of exon 25 and 26. In family DOA103 the deletion was also present in an affected male and his daughter. In family DOA105 the deletion also segregated with the disease as it was present in two affected and absent in two unaffected individuals (Figure 2A).

**Table 1:** Clinical data of ADOA patients with deletions in *OPA1*.

Patient	Age at diagnosis (y)	BCVA at diagnosis	Refraction, spherical equivalent	Color vision, Farnsworth Panel D-15	Visual Field	Disc and fundus appearance	VEP
DOA101	23	0.4/0.3	-6.5/-7.0	Dyschromatopsia, tritan axis	Normal outer boundaries, relative central scotoma	Temporal pallor	Borderline subnormal

DOA102	52	0.7/0.9	-3.5/-4.25			Temporal pallor	Pathological

DOA103	12	1.0/1.0 (0.2/0.2 at age 37 y)	-4.25/-3.5	Few errors on saturated, more on unsaturated, tritan axis	Centrocoecal scotoma OU, age 37 y	Small, evenly shaped disks	N/A

DOA104	28	0.4/0.2	+0.75/+1.0	No significant abnormality	Outer borders normal, mild relative central scotoma	Small, evenly shaped disks	Normal

DOA105	43	0.4/0.2	-3.75/-3.25	Tritan errors	Outer borders normal, mild relative central scotoma	Temporal pallor	Borderline subnormal

DOA106	52	0.5/0.5	plano/plano	N/A	N/A	Temporal pallor	N/A

DOA107	54	0.1/0.3	plano/-2.75	Dyschromatopsia, no specific axis	Outer borders normal	Atrophy, temporal pallor	Normal

DOA108	33	0.6/0.6	-1.25/-1.5	Mild trian-axis dyschromatopsia	Outer borders normal	Mild temporal pallor	Normal

DOA109	30	0.3/0.4	+4.0/+3.0	Dyschromatopsia, no specific axis	Outer borders normal, mild relative central scotoma	Temporal pallor and atrophy	Anomalous configuration

DOA110	16	0.3/0.3	-1.75/-1.25	Dyschromatopsia, no specific axis	Outer borders normal, mild relative central scotoma	Temporal pallor	Delayed implicit times

In family DOA106 we identified a deletion of exons 26 and 27 in the index patient. The deletion was also found in an affected daughter. A deletion of exon 28 was found in index patients from two independent families (DOA107 and DOA108). Only probe 06949-L06529 of P229 was deleted. This probe is located 104 nucleotides downstream of exon 28, and thus a manually designed MLPA probe located in intron 27 was made. As this probe was also deleted we conclude that exon 28 is deleted in these two families. In family DOA107 an affected individual had the deletion while an unaffected brother did not harbor the deletion. Family DOA108 is one of the oldest and largest known families with ADOA registered in Denmark with more than 28 affected and about 50 unaffected or unexamined individuals. Segregation analysis showed that the deletion segregated with the disease (Figure 2C).

## Discussion

The main finding of our study is that in a series of 40 unrelated ADOA patients *OPA1 *deletions were found in 10. The 40 patients were selected from a cohort of 100 unrelated ADAO patients of whom 60 were found to have mutations in *OPA1 *by sequence analysis (data not shown). Thus assuming that the 60 patients do not have further mutations in *OPA1*, we find a frequency of 10% of deletions in *OPA1 *in Danish ADOA patients. Notably, the two patients with complete deletions of *OPA1 *did not present with any other symptoms than classical ADOA, supporting that haploinsufficiency is the pathogenic mutational mechanism causing classical non-syndromic ADOA phenotype. Additional studies are needed to determine the extent of the deletion by mapping the deletion breakpoints, which is beyond the scope of this report.

Our study is an agreement with the report of Fuhrmann et al. [23] who showed that ADOA can be related to genomic rearrangements of *OPA1*. We found *OPA1 *deletions in 10 out of 40 patients selected from a cohort of 100 patients corresponding to a frequency of 10%, which is comparable to 12.9% found by Fuhrmann et al. [23], however, contrary to Fuhrmann et al. [23] we found no duplications. The rearrangements are scattered throughout the gene, affecting single or multiple exons, or even whole gene deletions. Furthermore, the deletions described in this study are different from the rearrangements found by Fuhrmann et al. [23], showing that they arise as separate events, rather than being hotspots for rearrangements. Therefore, we suggest that deletion and duplication analysis of *OPA1 *should be included in the routine genetic analysis of ADOA patients.

Several studies of various genes have shown that deletions or duplications not detectable with sequencing or screening strategies such as single strand conformational polymorphism (SSCP), contribute to the mutational load, which is confirmed by our study [20,24]. Thus, genomic rearrangements have to be considered in diseases where a proportion of patients apparently do not harbor mutations in the disease causing gene/genes, since these will be left unrecognized by sequence analysis which is the preferred method for mutation analysis. MLPA is a fast and relatively cheap method to analyze for gene dosage differences among large groups of patients.

## Conclusion

*OPA1 *genomic deletions account for about 10% of ADOA cases in a Danish population, registered at the national center for hereditary eye diseases. Our findings suggest that an analysis of genomic rearrangements is mandatory in the investigation and diagnosis of ADOA.

## Competing interests

The authors declare that they have no competing interests.

## Authors' contributions

All authors have read, edited and approved the final manuscript.

GJA performed experiments, analyzed data and drafted the manuscript. KG helped designed the study, analyzed data and drafting the manuscript. JE designed the study and experiments. KBN, ML, DM contributed to writing of the paper. ML and DM helped with the ophthalmological clinical information.

## Pre-publication history

The pre-publication history for this paper can be accessed here:

http://www.biomedcentral.com/1471-2350/12/49/prepub
